# Illuminating a joyless life: qualitative, transdiagnostic exploration of anhedonia

**DOI:** 10.1192/bjo.2026.12004

**Published:** 2026-06-01

**Authors:** Clementine J. Edwards, Chloe Osei-Cobbina, Alison Duerden, Nicola Sirey, Vidhi Bassi, Adeola Agunbiade, Matteo Cella, Barnaby D. Dunn, Andrew Gumley

**Affiliations:** Department of Psychology, Institute of Psychiatry, Psychology and Neuroscience, King’s College London, UK; South London and Maudsley NHS Foundation Trust, London, UK; Independent Researcher, Southampton, UK; Department of Psychology, University of Exeter, UK; Institute of Health and Wellbeing, University of Glasgow, UK

**Keywords:** Anhedonia, psychosis, depression, qualitative research, lived-experience involvement

## Abstract

**Background:**

Anhedonia (loss of pleasure) is a core feature of both depression and psychosis and yet the experience is not well understood. This limits our ability to effectively target it with psychological or pharmacological interventions.

**Aims:**

The aim of this study was to explore the experience of anhedonia, for the first time from a transdiagnostic perspective.

**Method:**

Semi-structured interviews, co-facilitated by lived-experience experts, were conducted among 17 adults with a diagnosis of depression or psychosis and who were experiencing anhedonia. Reflexive thematic analysis was employed to generate themes.

**Results:**

Six themes were identified: (a) no longer experiencing pleasure or joy in previously enjoyable activities; (b) grieving for the joyful times that have been missed; (c) the dilemma before trying an activity again; (d) the significant social impact of anhedonia, and the power of lived-experience connections; (e) uncertainty around what causes anhedonia; and (f) the lack of acknowledgement or support from services around this experience. The words disconnection and frustration were those most used to describe what people felt when experiencing anhedonia.

**Conclusions:**

The results highlight the negative impact of expectation and social pressure on joy, and the importance of the anticipatory period prior to trying an activity again. The clinical implications highlight the importance of discussing anhedonia with patients: by not doing so is contributing to stigma. This is the first study to directly explore anhedonia in adults, with lived-experience input throughout, and the findings support further work adopting a wider transdiagnostic approach.

Anhedonia is defined as the inability to feel pleasure during activities that were previously enjoyable. This experience is a core diagnostic feature of both depression and psychosis,^1^ and predicts transition to diagnosis in high-risk groups^2^ as well as worse outcomes for those who experience it.^3–5^ Anhedonia also contributes to a wider impact on well-being, defined as a person’s ability to experience meaning and form meaningful connections with others.^6^ Anhedonia is not effectively targeted by existing interventions.^7,8^ Loss of pleasure has been highlighted as a priority area for recovery by those with lived experience^9,10^ and their carers.^11^ Despite the clear importance of anhedonia as a predictor of outcomes and barrier to recovery, it is not well understood, which limits the development of effective treatments. Furthermore, the impact of anhedonia is not limited to psychosis and depression; it is a feature of a range of difficulties such as obsessive–compulsive disorder, post-traumatic stress, substance misuse, dementia and eating disorders.^12^ Phenomenological qualitative research is a method that enables us to explore and describe human experiences such as anhedonia, and to understand the meaning that people make around this phenomenon.^13^ To move the field forwards, we need to understand these perspectives – providing context to quantitative findings and identifying mechanisms to be examined in a research agenda for future work. The value of exploring joy through exploratory qualitative methods was highlighted recently in a study in UK adults.^14^ This work identified the huge impact that joy has on well-being and the intense personal significance of joyful moments, despite their fleeting nature. In clinical populations, only one qualitative study has explored anhedonia specifically through interviews conducted with adolescents either diagnosed with depression or experiencing elevated levels of depressive symptoms.^15^ Participants described a loss of joy during previously enjoyable activities, but their experience of anhedonia was much more than this, with all emotions feeling dampened, low motivation, disconnection, detachment and loss of identity being reported by participants. These findings provide key insights into the wider impact of anhedonia, with domains such as motivation and social withdrawal also identified by people with psychosis alongside feelings of numbness or emptiness in qualitative work exploring the broader construct of negative symptoms.^16–18^ A ‘bottom-up’ review, conducted in partnership between experts-by-experience and academics, highlights the impact of loss of pleasure in lived-experience accounts of depression more broadly.^9^ Qualitative studies conducted in adults have explored broader phenomena (depression, negative symptoms), with few specific insights into anhedonia or a disorder-specific focus. There are also very few examples of lived-experience input into study design, conduct or interpretation of findings. This limits the validity of the conclusions that can be drawn, and indeed may be contributing to the current lack of clarity and understanding around this experience, despite decades of research.

## Aims

The aim of this study is to explore the transdiagnostic experience of anhedonia, including in those with a depression and/or psychosis diagnosis, and to focus on the ‘in-the-moment’ processes as well as the wider impact, to increase our understanding of this experience. Individual qualitative interviews were co-designed and co-facilitated by people with lived experience to support in-depth, sensitive discussions. Thematic analysis was applied to reflexively understand the data,^19,20^ with representation of the range of perspectives included in the study.

The study objectives identified through discussion with the Lived Experience Advisory Panel (LEAP) were as follows:

- to describe what happens during activities that were previously enjoyable, for those people who are experiencing loss of pleasure in the context of depression and/or psychosis;
- to explore the experience of anticipated and remembered pleasure;
- to understand if/when these experiences are similar or different across different mental health conditions;
- to understand how people make sense of these difficulties and what meaning they attribute to them;
- to describe the impact of these difficulties on those who experience them (e.g. low motivation);
- to describe experiences, events or interactions that have made these difficulties worse;
- to share examples of things that have helped and ideas for support that might be helpful in the future.

## Method

In line with recommendations to implement open science practices in qualitative research, the procedure and topic guide were pre-registered on the Open Science Framework^21^ and are reported in line with the CoreQ Checklist.^22^

### Recruitment

The authors assert that all procedures contributing to this work comply with the ethical standards of the relevant national and institutional committees on human experimentation, and with the Helsinki Declaration of 1975 as revised in 2013. All procedures involving human patients were approved by the NHS HRA London-Central Research Ethics Committee (no. 24/LO/0578). Participants were recruited through self-referral following advertisement to the public through online platforms and email newsletters circulated by two UK lived-experience organisations: the McPin Foundation and National Survivor User Network. A second route for recruitment was through local South London & Maudsley (SLaM) NHS Foundation trust services; the study was advertised to clinicians who could refer patients on their caseload after asking for permission to share their details with the research team. The study was also advertised on the SLaM Take Part in Research web page, and potential participants could self-refer by responding to this advertisement.

Participants were screened to ensure that they met the following eligibility criteria.

#### Inclusion criteria

- Aged over 18 years;
- diagnosis of depressive disorder, bipolar disorder (current depressive episode) or a psychotic disorder;
- currently experiencing difficulties with loss of, or reduced, pleasure, as confirmed by a score of >1 on the Beck Depression Inventory-II^23^ items ‘loss of pleasure’ and ‘loss of interest’.

#### Exclusion criteria

- Does not have capacity to give informed consent;
- has a primary organic cause for their experiences of depression or psychosis (e.g. substance misuse, brain injury).

### Participants

Purposive sampling was employed to recruit a sample representing the relevant characteristics, specifically gender, ethnicity and diagnosis (representation of both depression and psychosis). This was done by first asking potential participants to complete a screening form, followed by the lead author surveying the responses to purposively select a representative sample for invitation to take part. There were no relationships established with participants prior to them taking part. A further 17 eligible people expressed an interest in taking part online and for whom we did not have capacity to include in the study, and a further 2 individuals who were approached following clinician referral declined to take part. Of note, and in line with a growing problem in qualitative research, many (20+) ‘fake’ participants expressed an interest via the SLaM Take Part in Research webpage when the study was launched; these were identified through some of the characteristics outlined in the recent editorial by Ridge and colleagues,^24^ and were not approached for checking for eligibility.

### Procedure

The interviews took place either online (Microsoft Teams) or in person at King’s College London, Denmark Hill Campus or a SLaM NHS setting; the participant’s preference was followed. The interviews were scheduled to last 1 h.

At the start of the interview, participants gave written informed consent for their participation and a brief questionnaire was completed, recording the following information:

- age
- gender
- ethnicity
- diagnosis on first contact with mental health services
- employment status.

The semi-structured interview was conducted, led by the LEAP member and co-facilitated by the lead researcher (C.J.E.). The topic guide was developed in close collaboration with the four LEAP members who co-facilitated the interviews. Participants were reimbursed £25, either through direct bank transfer or with an online voucher, for their participation in the study. The lead researcher made field notes alongside each interview.

The data were transcribed using Microsoft Teams, and these transcriptions were checked for accuracy and de-identified by the lead researcher.

### Lived-experience involvement and co-facilitation

This project is part of a wider programme of research that includes collaboration with a LEAP team comprising nine members. The LEAP members were approached with the opportunity to co-facilitate the interviews in this project, and four female LEAP members (representing a range of ethnicities, ages and mental health-related experiences) expressed an interest and took on this role. The lived-experience co-facilitators did not independently contact participants, or support with recruitment or informed consent process, which was conducted by the lead researcher. Their role was to lead the qualitative interview, supported by the co-produced topic guide, with the lead researcher always present to support facilitation. Two training sessions were offered for the co-facilitators (1 × 2 h, 1 × 1 h), with information on qualitative interview skills presented by the lead researcher and opportunities to role-play sections of the topic guide in pairs. The interviews were allocated to co-facilitators based on availability, and all four co-facilitators were able to conduct at least one qualitative interview during the study. Lived-experience co-facilitators attended a 15 min briefing before each interview and a 15 min debrief following each interview with the lead researcher; their reflections in these discussions were included in the field notes.

### Analysis

Reflexive thematic analysis^19^ was applied to the data-set to identify themes and subthemes that captured the depth and breadth of participants’ experiences. Quality criteria recommended for reflexive thematic analysis were followed during the analysis process,^25^ with NVivo software version 15 for Windows (Lumivero, Burlington, Massachusetts, USA; https://lumivero.com/products/nvivo/) used to support this. This is a deviation from the original pre-registration, which is detailed on the Open Science Framework and in the supplementary material. A detailed description of the approach, with examples of each stage, is also outlined in the Supplementary material.

### Researcher reflexivity

The team were led by C.J.E., who has 10+ years’ experience working with people with psychosis as a clinician, in addition to researching the topic of anhedonia, or loss of pleasure. C.J.E. was primarily clinically trained in cognitive–behavioural therapy (CBT), completed a PhD and further research from a critical realist standpoint and holds both these theoretical positions. The research team represents diversity in gender, age, socioeconomic background and ethnicity. C.J.E. was present as a co-facilitator for every interview, taking field notes while the interview was led by a lived-experience co-author. This reflective role allowed C.J.E. to consider any shared ideas or experiences with other interviews and any unique perspectives offered by the participant; given her knowledge of the research field, she also considered links with existing evidence. The briefing and debriefing discussions among co-facilitators allowed her to discuss these ideas with a LEAP member as data collection was ongoing. A more detailed reflexivity statement is available in the supplementary material.

## Results

### Participants

Information power was considered to determine the size of the sample,^26–28^ specifically the depth and breadth of the data-set. Team discussions were held at regular intervals during the data collection period, initial themes identified were shared across interviewers (C.J.E. was present for all interviews) and the ability to address the research questions was considered. After 13 interviews it was felt that more perspectives from men, Black people and those with a psychosis diagnosis would improve the information power (breadth and depth of the data-set), and these characteristics were sought in the four subsequent interviews. Following the completion of these interviews, it was felt that early themes had been supported in these additional data, and that there was sufficient information power to address our research questions; recruitment was stopped at this point.

Of the 17 participants, 9 (53%) were female and 8 (47%) were male. The average age of the sample was 44.5 years (s.d. 13.7, range 21–65 years) and the mean length of time that people had been in contact with mental health services was 23.8 years (s.d. 12.8, range 0.5–42 years). The majority of the sample were employed, 25% full-time and 29% part-time; participants were either retired (*n* = 2), self-employed (*n* = 1) or a student (*n* = 1), and 18% were currently unemployed. The majority of the sample were White (59%); other ethnicities represented were Black British or African (*n* = 3), Chinese (*n* = 1), Bangladeshi (*n* = 1) and mixed (*n* = 2). A range of diagnoses were represented: 59% of participants had a diagnosis of depression (two of these participants reported a diagnosis of bipolar disorder) and 35% had a diagnosis of psychosis, with one participant reporting both these diagnoses. It is noteworthy that many participants reported additional diagnoses, including obsessive–compulsive disorder (*n* = 4), post-traumatic stress disorder (*n* = 3) and emotionally unstable personality disorder or borderline personality disorder (*n* = 3).

All participants were attributed pseudonyms in line with guidance from the LEAP members, to maximise relatability and accessibility of the reporting.

### Findings

The initial coding process conducted by the lead researcher resulted in the generation of 17 subthemes, which were grouped into 4 broader themes (see hierarchy chart in Appendix). The first two discussions with the wider research team, including LEAP members, identified areas where there was overlap and repetition, and therefore reducing the number of subthemes was recommended. Through an iterative process, these 17 subthemes were refined into the 6 themes that will be described below. Quotes will be attributed to pseudonyms, with D denoting where the primary diagnosis was depression and SZ when it was schizophrenia spectrum disorder.

### Theme 1: anhedonia is a profound loss of pleasure in things you used to enjoy

Across all the interviews, people described noticing anhedonia when they try to do something they previously enjoyed and no longer enjoy it: ‘I love watching soccer also, so we could be watching a soccer game and I’d just be staring at the game with a blank stare, just not actually enjoying it.’ (Obafemi, D)

**Fig. 1:**
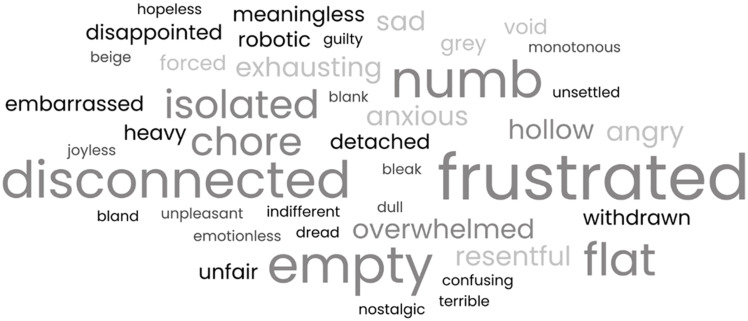
Word cloud representing all the words used by participants to describe how they feel when participating in an activity they used to enjoy but are now experiencing anhedonia. The size of each word represents the frequency at which it is present in the transcripts: larger words were used more often.

Participants used a wide range of words to describe how they felt during these activities, rather than enjoyment or pleasure. These are summarised in the word cloud in Fig. 1. Notably, there are relatively few uses of words such as sad or low, and many more descriptions of feeling disconnected, numb or empty. Some examples of these descriptions in context are included in the quotes below:

‘I’m just going through the motions and kind of my mind’s kind of blank.’ (Hassan, SZ)

‘It’s quite mild when I’m alone. OK, so I don’t really feel like that. It’s not that concentrated, but when I’m around people, and particularly around my group of friends, that is people we enjoy doing stuff together, so it sort of shoots up to a very, very, very pronounced level. Like I sort of feel this emptiness, this barrenness within.’ (Obafemi, D)

Participants did not describe anhedonia as a constant experience, with many commenting that sometimes they were surprised by enjoying an activity or noticing a period where it improved. However, for many people it had been a problem for a long time, Leanne (D) described this unpredictability: ‘Maybe throughout a month. There’s like a day or two days or something’s different, but I’d say quite persistently for quite a long time. And I enjoy those times that are different, but I can’t predict when they’re going to come.’

### Theme 2: grieving the lost self

Understandably, participants felt a profound sense of loss of all the enjoyable times they had missed out on over the years they had been experiencing anhedonia: ‘I mean I get tearful most evenings because I’m not enjoying stuff. I just feel like I’ve missed out on so much’ (Liz, SZ). In response to this, many described feeling frustration, and anger, that this had happened in the first place, and that despite a huge amount of effort things were still not enjoyable: ‘Then I feel angry. You know that I’m putting all this effort in [sic] but not getting any enjoyment back.’ (Zoe, D)

As well as experiencing anger and frustration, a substantial minority of people reported blaming themselves and thinking a lot of self-critical thoughts when they experience anhedonia:

‘I’m putting up such high expectations for myself, and I won’t be able to achieve those now. Because it makes me feel in a way of failure.’ (Ali, D)

‘Why aren’t I enjoying it? What’s wrong with me that I’m not enjoying it? Am I a freak? Am I all the negative things I could be?’ (Teresa, SZ)

Leanne (D) acknowledged that it can be upsetting to think about previous times, but went on to say that remembering previous joyful times gave her hope that she can experience those feelings again: ‘Yeah, I guess those are the times that give me hope that things can be different. I can enjoy things again or like that. Still, like physiologically, I can still do that. I think yeah, those times are really important.’

### Theme 3: should I try again? The constant dilemma when there is no joy

Making the decision to try an enjoyable activity again is a dilemma when you are experiencing anhedonia. Many participants described knowing that it would be beneficial for them to try again, and yet motivation is a real challenge, as described by Adam (D): ‘But if you’re, if you’re feeling really low, you just don’t have the energy and you don’t. You don’t actually want to do it even though you kind of know in the back of your head it’s good for you. But it’s such a struggle, you know, to to kind of come up and to motivate yourself, you know, to do something.’ Faisal (D) explained that it is difficult to know what will help you feel better or worse, and many participants echoed his words of being stuck between a rock and a hard place: ‘But then the alternative is, you know, it’s like, well, if I don’t go out and go for a walk, then I’ll be stuck at home. And then I’ll even feel worse about myself then that way. So you know, you’re stuck in a in a place like up between a hard place and a rock like, you know, kind of like, well, what do I do now?’

This dilemma is present whenever an activity presents itself, but participants described how it is particularly intense in the days prior to a planned enjoyable activity: ‘This should be great. I should enjoy it. It should be nice. It should be all of those things. And I’m dreading it’ (Teresa, SZ). Sometimes people manage to go to an activity by removing the pressure on enjoying it and instead focusing on a different reason to do it. Hassan (SZ) mentioned a sense of achievement ‘I’ll go with the hope of you know having a sense of accomplishment and stuff like that coming out of there and you know, at least putting in the effort.’ Liz (SZ) identified valuing a connection with friends: ‘I’m trying to tell myself the reason you want to do this is because you want to see your friends and you want to have a life*.’* Other reasons mentioned included exercise, physical health, fulfilling a family role/duty and helping others.

### Theme 4: anhedonia is isolating but it is helpful to connect with others who experience it

The pressure to enjoy activities isn’t just an internal one; many people spoke about feeling the expectation from others to experience pleasure when spending time with them and then feeling very disconnected when they were experiencing anhedonia: ‘It makes me feel more isolated. Like oh, I can’t share my thoughts or feelings with these people because they won’t understand what I’m going through’ (Faisal, D). The lack of awareness or understanding of anhedonia as an experience has made this harder; almost everyone we interviewed said it was the first time they had spoken about it, or even knew that it had a name: ‘I know labels aren’t always the best things, but I think if there is a name you feel, oh, it’s recognised, then it’s actually, you know, it is recognised and people it’s not just me that has it’ (Sarah, D).

When partcipating in social activities, many participants reported feeling forced to pretend that they were enjoying these, describing how tiring it is to have to wear a mask of enjoyment and worrying that they can’t keep it up and that people will see that they are pretending or not enjoying spending time with them: ‘It’s not like I don’t love my friends and want to see them it. It’s this thing of having to mask it all the time’ (Liz, SZ). Others described feeling as if anhedonia had reduced their interests so much that they would not have anything interesting to talk about: ‘I also feel like in that loss of pleasure I feel like I’ve not got anything interesting to say’ (Ian, D). These two perceived pressures – to be interesting and to enjoy things – are understandably often too much and participants described avoiding social events as a result, with a huge negative impact on their social network, as Ian went on to explain: ‘So again, that loss of pleasure thing is sometimes either avoid that social situation altogether or if I’m there is having to mask as if to say like, yeah, I’m having a great time’ (Ian, D).

Participants told us that, although they found it very difficult to share with many of their family, friends and colleagues, they could talk to other people they knew who had experienced anhedonia or had other challenges with their mental health. This was a vital source of support for many of the people we interviewed: ‘And what was a great comfort to me was to know that I had a small circle of fellow service users’ (Paul, D). Participants also found it very positive to be interviewed by a lived-experience co-facilitator and said they found this helped them feel understood: ‘I’m relieved in a way. That I’ve had Interviewer 1 who I can be honest with, and who also has lived experience of it. So it’s been good to be interviewed by somebody with lived experience’ (Teresa, SZ).

### Theme 5: the loss of pleasure is unexplained

Overall, participants described being unsure what had caused their anhedonia, with people presenting only tentative explanations for what they felt accounted for their experiences. Some people interpreted it as being linked to their wider mental health challenges, such as psychosis, as described here: ‘When I’m not particularly in touch with reality. It can feel like there’s an entity that’s if you like, got it in for me. That is stealing all my pleasure. Taking it away’ (Teresa SZ); or depression: ‘It’s so hard just to pinpoint it on the anhedonia. It’s all kind of interlinked with the depression, depressive episodes’ (Cynthia, D).

However, some participants noted that it can occur or persist when other difficulties have improved: ‘There have been times that everything else was mentally better and it’s been I’ve experienced it on its own and that’s almost been more weird because it’s strange. Feel like everything’s right and in place and I’m not experiencing what I should be’ (Jasmine, D).

Stressful life events were noted as a trigger, as well as changes in psychiatric medication, with some people, including Sarah (D) below, reflecting on whether antidepressant or antipsychotic medication had impacted on their ability to feel emotions: ‘But I just wonder whether coming off drugs would make me feel joy again, and but maybe you’ve got to take the rough with the smooth.’

### Theme 6: anhedonia? Heal thyself

Almost every participant said that the study interview was the first time they had discussed their experience of anhedonia. Most people had not heard the word before they saw the study advert, despite it being something they had experienced for a long time. Participants specifically reported very few, and often a total absence, of direct conversations about it with mental health professionals as part of their care, including in psychological therapy: ‘But no one ever sat down and said this is quite a significant symptom in your illness and this is, you know, something we’d like to look into’ (Gary, D).

Participants described interactions with mental health clinicians about the impact of anhedonia, particularly focused on planning activities, which they felt were unhelpful because they did not directly address the anhedonia itself. Leanne describes this experience in the following quote: ‘There can be long lists presented to you of things that you could try to enjoy or do. And I think at that point I switch off because I feel like there’s a real lack of understanding. That it doesn’t feel quite as straightforward as just trying a few things off the list. I’ve like tried for a long time, on and off for a lot of years to find things that I enjoy, and I don’t. So, giving me a list, age 40 is is probably not going to cut it’ (Leanne, D).

Many participants mentioned this in relation to their experience of CBT, the National Institute for Health and Care Excellence-recommended psychological therapy for many populations where anhedonia is common, including psychosis and depression:

‘But I never really got around to talking about potential tools and strategies and solutions to help me. In terms of, you know, trying to do pleasurable stuff, but obviously if you’ve got anhedonia the pleasurable stuff, you know, you don’t really get the same level of pleasure from. So, what do you do?’ (Adam, D)

‘I had a brief course of CBT to try and change my thought pattern and behaviour, but it didn’t change how I felt in myself.’ (Teresa, SZ)

Participants had tried lots of things to improve their enjoyment of activities, without support from mental health services. For some, sticking to a routine was really helpful: ‘Having a routine, for example exercising and doing this that, but although you don’t get enjoyment out of it you, you feel accomplished’ (Hassan, SZ). Others found that being open to new experiences helped them to find enjoyment: ‘So sometimes it’s worth just trying something you don’t normally do. If you’re struggling, and then it seems to put a bit of a light bulb on’. (Zoe, D)

Many people described moments when they noticed a small pleasure that was sparking a feeling of joy, and trying to be present when this happened was helpful:

‘And you can’t keep cancelling everything and you’ve got to try and get some enjoyment out of it, even if it’s just the little things like. Oh, that’s a nice scenery, you know, or. Oh, look at this. You know, teapot or something and you’re out or something. Enjoy the. Enjoy the food.’ (Liz, SZ)

‘Sometimes when I watch a comedy, you know, I am able to smile or laugh at certain jokes or whatever.’ (Adam, D)

‘And when I’m there, I try to be fully present, So I try to focus on the smell of the flowers, the shape of the leaves. The you know how fast the water is going and in a way that sort of brings me peace because. Even though I’m not at the best, I try to find enjoyment in nature.’ (Faisal, D)

Although most participants saw anhedonia as something they hoped to overcome, some people commented that accepting they might not enjoy things as much as other people, or they did in the past, helped them to try activities again: ‘And like this is who I am and how I’m wired and learning to live with that’ (Toby, D).

## Discussion

The aim of this study was to explore the subjective experiences of anhedonia in people with psychosis and affective disorders. This was done in partnership with experts-by-experience, who shaped every stage of the research process.

Our study found that people notice anhedonia when they are trying to do things they used to enjoy, particularly when doing these activities with other people. The social impact is significant, and the exception to this was connecting with others with lived experience of mental health problems. The trajectories of anhedonia varied across participants; sometimes anhedonia persisted in the absence of other mental health challenges and for others it was linked to the worsening of other difficulties (e.g. anxiety, low mood, paranoia). It was clear that anhedonia is not a stable experience but can relent unexpectedly at certain moments. Many participants had not spoken about anhedonia before, despite engaging in psychological therapy and receiving care from mental health services. Solutions and tips for managing anhedonia were described by participants who had persisted in trying to find joy, often for many years, without support from services.

Our findings support a transdiagnostic and transdisciplinary conceptualisation of anhedonia, in line with previous research.^15^ Anhedonia cuts across multiple domains including environmental context, internal psychological processes (e.g. identity, beliefs) and existential or spiritual experience (e.g. meaning, purpose).^14^ The dynamic nature of anhedonia was strongly expressed in our study, and this further highlights the limits of retrospective self-report in this field, which was also a finding in the previous work. Our findings also describe an intense decision-making process that happens in the anticipation phase, before engaging in something you might enjoy. This goes beyond not looking forward to something (i.e. an anticipatory pleasure deficit) and suggests that anxiety about the recurrence of anhedonia occurs prior to an event or activity, which may result in avoiding the potentially enjoyable activity, either by not participating or experientially avoiding pleasure or joy if they do. The description of this dilemma links to the idea that anhedonia may be the result of a reduction in sensitivity of the behavioural approach system and a corresponding increase in sensitivity to behavioural inhibition processes. This idea has been explored across psychosis and depression, with emerging findings supporting links between changes in sensitivity and anhedonia, although further work is needed that overcomes the limits of self-report measures of these constructs.^29,30^ Reduced engagement or presence during previously enjoyable activities was also identified by participants in this study as a potential mechanism underlying anhedonia. Participants described either a totally empty, or blank, mind during an activity, or one that was very busy with negative thoughts, some of which could be described as ‘positive dampening appraisals’ (e.g. ‘this isn’t as good as it used to be’)^31^ or social concerns such as people noticing they were not enjoying it. Overall, these findings suggest factors that impact on pleasure both during an activity (consummatory pleasure) and prior to it occurring (anticipatory pleasure) rather than a specific deficit in one of these constructs.^32^

Some of the themes in the current study are echoed in the ‘bottom-up’ systematic review of the lived experience of depression:^9^ specifically, feelings of numbness and detachment, difficult decision-making, social isolation and disconnection and the tiring experience of needing to pretend or ‘put on a mask’ when with others. The identification of themes around disconnection, numbness and detachment, and their significant representation in the word cloud, contrast with the low prevalence of words such as hopeless or sad, reinforcing the separation of negative affect (sadness, low mood) and anhedonia. The findings of the current study suggest that anhedonia, a key symptom of depression, is contributing to many of the aspects of the wider experience described in the lived experience accounts across the field.

Many words describing difficult, negative emotions are present in participants’ accounts, including shame, guilt, loss and fear. These can be understood as self-stigma, an internalised negative view of self, following experiences of external stigma and discrimination, that is prevalent among people with mental health disorders, including psychosis and depression.^33–35^ Many of our participants spoke about their fear of negative social evaluations and feelings of shame or guilt, leading them to hide their experiences of anhedonia. Several participants described a cumulative process involving repeatedly trying to enjoy things, and not doing so, became interpreted as evidence that there was something wrong with them or as a sign of personal failure, in the absence of an understanding of anhedonia. This maintains self-stigma, which then impacts on well-being and self-esteem. It is notable that, although meaningful social connections with others are a protective factor for self-stigma, interpersonal effectiveness is reduced in the context of shame and guilt,^36^ and our participants highlighted the fact that social relationships are significantly impacted by anhedonia.

The participants also spoke about how losing joy in activities that were enjoyable in the past disrupted their sense of self and identity. A disrupted sense of self, and body, is common in psychosis^37^ and depression^38^ and can impact perception and sensory experiences. Our interviews suggest that the experience of anhedonia contributes to a distorted perception of the world, with reduced capacity to sense and perceive what is happening around one. This experience of altered perception is common in trauma-related distress and impacts on functioning.^39^ It is reflected in this paper through language such as ‘numbness’ and ‘disconnected’ echoed by many participants, who also spoke about reconnecting to sensory experiences such as nature as a promising avenue for recovery.

The findings suggest several important avenues for future research: first, to build upon this work to understand the cognitive processes happening before and during potentially enjoyable activities in transdiagnostic quantitative studies. Progress has been made in examining positive dampening appraisals using self-report measures,^40^ although not in psychotic disorders, and a new scale (Leuven Exeter Dampening Scale) has recently been validated to improve measurement of these beliefs.^41^ Using experimental frameworks that allow the examination of beliefs in-the-moment (e.g. Dunn et al^42^) would be a useful approach in the further exploration of this potential mechanism. The findings of our study suggest that the thoughts people have during the anticipation phase, which may extend over several days before the activity, are important, particularly fear of anhedonia recurrence and how this may link to avoidance. This is a relatively unexplored target mechanism, although studies have identified links between depression and fear of happiness or positive evaluation (praise).^43,44^ The wide range of comorbid diagnoses represented in this study calls for work to be conducted, including more diagnostic groups where anhedonia has been identified (i.e. eating disorders, substance misuse, post-traumatic stress disorder). Stressful life events, trauma and psychotropic medication were felt by some participants to contribute to anhedonia, but this view was not strongly held across the interviews and further work is needed to establish causal pathways. The lived-experience experts in the study team felt that further qualitative work should explore how anhedonia may be interpreted by those experiencing it in the context of other mental health difficulties, such as hearing distressing voices, guilt, low self-esteem and paranoia.

The study proposes three important clinical implications. First, that clinicians must discuss anhedonia with their patients; this was identified by participants as helpful in itself, and that not doing so is contributing to stigma and social isolation. These discussions should be trauma-informed and transdisciplinary, with anhedonia viewed in the context of the person’s environment, mental health and identity.^45^ Second, the findings echo qualitative evaluations of positive affect-focused treatments developed in depression, such as Augmented Depression Therapy,^46,47^ in which participants highlighted both engagement in activities that tap into small pleasures (e.g. nature walk, cup of tea, hot shower and mindful presence), or savouring, during these activities to counter positive dampening processes and promote enjoyment. The current study suggests that the application of these strategies may be beneficial for people experiencing anhedonia across diagnoses, although further work is needed in this area. Finally, the importance of peer support networks and spaces for people experiencing mental health difficulties was highlighted throughout the interviews in this study.

### Strengths and limitations

The key strength of the study is the involvement of individuals with lived experience at every stage, including those conducting the interviews. Many participants said that this strengthened their ability to be open in the discussions, and it enhanced the information power of the interviews. The reflexive thematic analysis was also strengthened by lived-experience experts, offering the opportunity for member-checking throughout the process, and this was also done directly with the participants. Although the sample was diverse in the representation of diagnoses, gender and ethnicity, most participants accessed the interviews online and the majority of the sample were employed, suggesting that those who were unemployed and/or had low digital literacy or accessibility are less well represented. There are also other important characteristics not captured, and so their representation is unclear (e.g. socioeconomic status). The transdisciplinary nature of anhedonia described in the findings highlights the limited disciplines represented in the authorship team, and we acknowledge the limitations this places on our interpretation of the findings, which was primarily through a lived-experience, clinical psychology and cognitive–behavioural framework.

Our study showed that anhedonia is an experience of disconnection and detachment, not sadness, and that it fluctuates, sometimes from moment to moment. Potential mechanisms identified include cognitive and emotional barriers to engaging in activities and enjoying them, both in advance of the event and during it. The participants had a lot of shared experiences, leading to the conclusion that anhedonia has many transdiagnostic features. Clinicians need to discuss anhedonia openly with people experiencing mental health difficulties, and treatments focusing on effective scheduling and mindful presence during activities may be beneficial across diagnoses.

## Supporting information

10.1192/bjo.2026.12004.sm001 Edwards et al. supplementary material Edwards et al. supplementary material

## Data Availability

The data that support the findings of this study are available on request from the corresponding author, C.J.E. The data are not publicly available due to their containing information that could compromise the privacy of research participants.

## Supplementary material

The supplementary material is available online at https://doi.org/10.1192/bjo.2026.12004

## Research material availability

All materials supporting the conduct of this study can be found in the supplementary material and on the Open Science Framework (doi:10.17605/OSF.IO/GQV2D).
