# Supplementary material for: Illuminating a joyless life: qualitative, transdiagnostic exploration of anhedonia
Source: BJPsych Open. 2026 Jun 1;12(4):e149. doi: 10.1192/bjo.2026.12004 (PMC13237115; doi:10.1192/bjo.2026.12004)
Supplement: Edwards et al. supplementary material [file S2056472426120043sup001.docx]

Supplementary Material

Interview Topic Guide

Illuminating a Joyless Life: A Qualitative Study Exploring Anhedonia

Individual Qualitative Interview Topic Guide

[Before starting the recording]

- Facilitators to introduce themselves.
- Aims of the project – This is the first project at the start of a 5yr programme of research [funded by the Wellcome] to understand anhedonia across different mental health diagnoses. The findings from this project will inform the studies that follow.
- Review the participant information sheet to highlight key pieces of information and check understanding (participants will have received this at least 24hrs prior to the meeting)
- Sign consent form (give them a copy)
- Plan for session – informal discussion, some questions from me but mostly want to hear your experience, no right or wrong answer, recording and transcript, limits of confidentiality – best person to contact if concerned (make a note of this), might note something down if I want to ask you some more about it.
- Interview should last approximately one hour (can stop at any time, let me know if you need a short break).

[start the recording]

[Questionnaire]

Some things that can get in the way of enjoyment are worry, pain, low energy or feeling sad. We are particularly interested in a different experience which is a loss of pleasure or enjoyment - not having a sense of pleasure or joy when you are doing something you used to enjoy. The focus of the interview will be on this experience; do you have any questions about what I mean?

1. So, we are meeting today because you shared with me that loss of pleasure, or enjoyment, is something you are experiencing at the moment? Is that right? [note participants’ language and perhaps use this as you go through].
2. When did you first experience a loss of pleasure or enjoyment?
   1. What was going on for you at that time?
   2. So, how long have you been experiencing this?
3. Could you tell me a bit about, what is it like to experience a loss of pleasure or enjoyment now?
   1. When do you notice it?
   2. How does it make you feel?
   3. Is it linked to other parts of your mental health?
   4. What is the impact of losing pleasure on your life?
      1. What is the social impact?
   5. What does it mean to you to experience loss of pleasure? How do you make sense of this?
4. What happens now when you are in an activity or event that you used to enjoy [Can I ask you to close your eyes if you feel comfortable and take yourself back to one of these times recently if it helps you to answer the question].
   1. What happens in your mind?
   2. What happens in your body?
   3. How does that make you feel?
   4. What does it mean to you?
5. How do you feel about going to or trying an activity you used to enjoy?
   1. How about the day before? And in the hours before?
6. Have you noticed anything that makes it harder to enjoy things? Anything that seems to help?
   1. How does this make it harder/help?
   2. Is there anything your friends and family could do to help?
7. What is it like to remember times in your life when you were enjoying things?
8. What support have you been offered around the loss of pleasure or enjoyment?
   1. Have mental health services offered any support with this experience?
   2. What has been helpful from services? What could they have done better?
9. What should be a priority for future research in this area?
   1. Why are these things important to you?

[Closing]

1. I’ve asked all my questions about this experience; did you have anything else you would like to tell me?
2. Do you have any questions for me?

[Turn off recorder]

- Thank you.
- Check in – how are they feeling after the interview?
- Voucher
- Explain what happens next – interviewing several people to hear about a range of experiences, will produce a transcript of each interview and then identify themes which will be written up for publication to share with more researchers and clinicians.

# Analysis

## Researcher Reflexivity

## An example of the impact of this was the early sense that everyone was talking about the social impact of anhedonia, interviewers then followed up in future interviews to ask about this specifically. The debrief discussions often focused on the delicate balance of self-disclosure for the interviewers with lived experience, sometimes they were concerned they had brought their own perspective into the discussion instead of giving the person space to share theirs. As co-facilitator CE was able to feedback any examples where she felt this might have occurred, but these were very rare, and did not occur in the majority of interviews, the instances of self-disclosure the lived experience co-facilitators made seemed to foster relatability and openness in the discussions and we felt added to the depth and breadth of the data being collected. Authors AG, BD and MC are experienced researchers in the field, they contributed to the design of the study and have reviewed the write-up.

The discussions throughout this project from design to write-up have therefore been conducted in groups with a range of lived experiences of the topic (researcher, clinician, service user, patient) and on many occasions different interpretations and perspectives were offered. When this has arisen, we have reflected on our positions in the team, aware of potential power differentials (e.g. supervisor-junior, lived experience expert – academic expert), and the importance of giving all voices equal weight. All the team brought experiences of anhedonia from outside of the interview to the discussions, this included direct lived experience but also that acquired through clinical and research roles – we reflected on the power of the experience in our team, using this to fuel our discussions and generate themes whilst returning to the transcripts to ground this in the data. The information power in the data was apparent when reviewing the final themes and a shared reflection of how sad it made people feel, in the core team, the wider LEAP and the participants who were contacted. This led to a desire to amplify stories of recovery and hope, but when returning to the data it became clear that whilst moments of joy were described, the content is focused on the challenges people have faced, rather than solutions. Some team members reflected that this could be due to the topic guide being more focused on the difficulties, while others thought it might reflect the lack of help offered to people experiencing anhedonia.

## Analysis Approach

The transcripts were read initially to check for accuracy, and then again to identify initial codes and related quotes. This involved reviewing one transcript at a time, alongside the field notes, and highlighting any quotes directly related to the participant’s experience of anhedonia, then adding them to either an existing code which had been previously generated or creating a new one to capture what was reflected in the quote. NVivo ^25^ was used to support this process. These codes were then collated into themes, where meanings were overlapping or repeated then codes were combined. Some codes only contained quotes from 1 or 2 participants, and these were combined with others to represent a greater breath of experience. Following this process, the codes were reviewed again – this time outside of the software, by writing them out in a visual representation. A hierarchy of themes and sub-themes was mapped, the focus was on doing this reflexively, with themes representing the meaning in the initial codes. These stages were all completed by the lead researcher (CE), who was also present during all the qualitative interviews.

The themes, sub-themes and quotes were shared with another researcher in the team (COC) and the four LEAP members who conducted the interviews. These were then discussed as a group through a series of 7 meetings as a small group and 1 with the wider LEAP, one LEAP member also shared written reflections. The wording of themes was altered, and quotes were selected to reflect these. The themes, sub-themes and quotes identified at the end of this process were then sent to the study participants for sense-checking (participants all gave their permission to be contacted for this purpose), they were asked to share their thoughts on whether these ideas captured the experiences and perspectives they shared with the researchers during the interview. This feedback was discussed with the wider research team, including LEAP members and incorporated into the wording of the themes.

Stages of Analysis

1. Example of transcript with initial codes highlighted (exported from NVivo software)

**QID001**12:51
I think that that also makes it worse, because then it makes me feel more isolated. Like oh, I can't share my thoughts or feelings with these people because they won't understand what I'm going through.
And so I find myself more socially withdrawing as well.

**Interviewer 1**13:04
Yeah, yeah, definitely.
And how do you how do you actually make sense of this?
Do you like? How do you make sense of how you feel like?

**QID001**13:15
I think what helped me get through some things is like I listen to podcasts based on positive psychology and, you know, mental health and, you know, sort of being like the Co-creators in your life and being an active agent in your own recovery.

**Interviewer 1**13:25
Yeah.
Yeah.

**QID001**13:34
And I find that.
In a way like a coping strategy.
Is possibly.
Going into, well for me, it's always been in nature. I think that really helps me going for a walk or just spending time outside like I find that when I'm go on a walk, I tend to go towards somewhere where it's sort of isolated and towards the forest.

**Interviewer 1**13:50
Yeah.

**QID001**14:00
And rivers. And when I'm there, I try to be fully present.
So I try to focus on the smell of the flowers, the shape of the leaves.

**Interviewer 1**14:07
OK.

**QID001**14:10
The you know how fast the water is going and in a way that sort of brings me peace because.
Even though I'm not at the best, I try to find enjoyment in nature.
Where, you know, like even if I'm not happy there still some serenity somewhere and that sort of helped me sort of be reflective in a way to see, you know what? What am I currently doing and what can I do to make myself feel better so I know.

1. Initial 21 codes generated by lead researcher (CE).

| 1 | The cause of anhedonia is unclear to the people who experience it. |
| --- | --- |
| 2 | Significant life transitions and events can trigger anhedonia. |
| 3 | People wonder if taking psychiatric medications contribute to anhedonia being worse or better. |
| 4 | Further research should focus on what causes anhedonia and early intervention |
| 5 | Anhedonia is viewed as a symptom, and is worse when overall mental health is worse. |
| 6 | It is really hard to do enjoyable activities when you are experiencing anhedonia. |
| 7 | When someone is doing an activity, and the pleasure is gone, they feel overwhelmed or empty. |
| 8 | People with anhedonia experience positive and difficult thoughts and feelings before a planned activity they used to enjoy, this makes it difficult to do. |
| 9 | People first notice anhedonia early in life and then it goes up and down over time. |
| 10 | Anhedonia is engaging in an activity that used to bring enjoyment, and not feeling any joy at all. |
| 11 | Helpful strategies have been identified through trial and error, and with little direct support from services. |
| 12 | Sticking to a routine and being open to novel experiences are both helpful approaches to activity |
| 13 | It is helpful when services directly address anhedonia, but this doesn't happen often. |
| 14 | Being present, particularly in nature, can bring peace and sometimes even joy. |
| 15 | Being able to share the experience of anhedonia with others, particularly those with lived experience is really important for recovery |
| 16 | Acceptance, sense of purpose and self-compassion help people engage with activities despite anhedonia |
| 17 | Anhedonia negatively impacts on many areas of life, particularly connection to others. |
| 18 | People experiencing anhedonia report a profound sense of loss, and anger. |
| 19 | Memories of enjoyable activities bring sadness and hope. |
| 20 | It is very difficult to share the experience of anhedonia with others, this leads to isolation and disconnection. |
| 21 | Anhedonia takes away important coping strategies for other mental health problems |

1. These codes were reviewed, those which only included 1 or 2 quotes from participants were combined with others to form new codes and the initial 17 codes (grouped into four themes) sent to the wider team were:

| **Theme 1: The cause of anhedonia is unclear to the people who experience it.** |
| --- |
| Significant life transitions and events can trigger anhedonia. |
| People wonder if taking psychiatric medications contribute to anhedonia being worse or better. |
| Further research should focus on what causes anhedonia and early intervention |
| Anhedonia is viewed as a symptom and is worse when overall mental health is worse. |
|  |
| **Theme 2: It is really hard to do enjoyable activities when you are experiencing anhedonia.** |
| When someone is doing an activity, and the pleasure is gone, they feel overwhelmed or empty. |
| People with anhedonia experience positive and difficult thoughts and feelings before a planned activity they used to enjoy, this makes it difficult to do. |
| People first notice anhedonia early in life and then it goes up and down over time. |
| Anhedonia is engaging in an activity that used to bring enjoyment and not feeling any joy at all. |
|  |
| **Theme 3: Helpful strategies have been identified through trial and error, and with little direct support from services.** |
| Sticking to a routine and being open to novel experiences are both helpful approaches to activity |
| It is helpful when services directly address anhedonia, but this doesn't happen often. |
| Being present, particularly in nature, can bring peace and sometimes even joy. |
| Being able to share the experience of anhedonia with others, particularly those with lived experience is really important for recovery |
| Acceptance, sense of purpose and self-compassion help people engage with activities despite anhedonia |
|  |
| **Theme 4: Anhedonia negatively impacts on many areas of life, particularly connection to others.** |
| People experiencing anhedonia report a profound sense of loss, and anger. |
| Memories of enjoyable activities bring sadness and hope. |
| It is very difficult to share the experience of anhedonia with others, this leads to isolation and disconnection. |
| Anhedonia takes away important coping strategies for other mental health problems |

1.
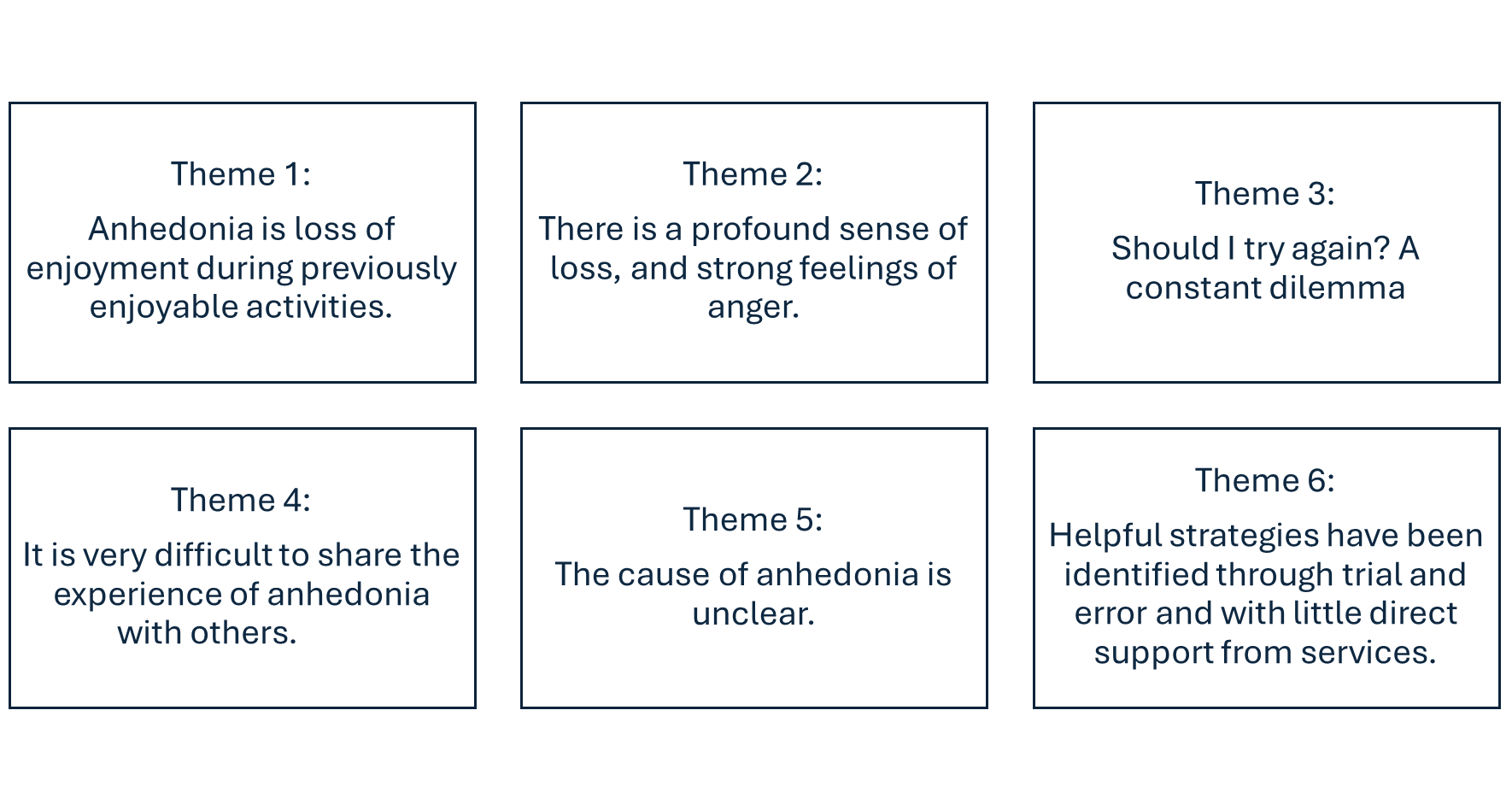
This was discussed with the research team (two researchers and four experts by experience) and it was felt to be too many themes, very difficult to extract meaning from and overly detailed. We discussed our perspectives on the key messages from the data in a further two meetings, and the lead researcher took this away and then proposed six revised themes.
2. Through three subsequent meetings the process of defining and naming the themes, and selecting quotes to illustrate them was done as a research team, resulting in the following six themes as presented in the manuscript:
3. These themes, and the selected supporting quotes were presented to the wider project LEAP (9 members) and emailed to all the participants who took part in the study for feedback, they did not suggest any changes to the theme labels and confirmed it represented their experience and what they shared in the interviews.

## Deviations from Pre-Registration

Open Science Framework pre-registration was updated on 07/01/26 to reflect the change to analytic approach from original pre-registration which stated Interpretative Phenomenological Analysis and template analysis would be applied. As interviews progressed, the lead author, in discussion with the rest of the authorship team elected to adopt reflexive thematic analysis as the analytic approach. This was to better capture the wide range of experiences and perspectives described in the interviews, as a foundational paper in a topic area with no existing research.
